# Supplementary material for: The impact of the COVID-19 pandemic for children in custody: An analysis of inspection reports
Source: PLoS One. 2024 Jun 20;19(6):e0304862. doi: 10.1371/journal.pone.0304862 (PMC11189207; doi:10.1371/journal.pone.0304862)
Supplement: S1 File — (DOCX) [file pone.0304862.s001.docx]

# Supporting Information 1

## During COVID Inspection Reports

<https://www.justiceinspectorates.gov.uk/hmiprisons/inspections/hmyoi-cookham-wood-7/>

<https://www.justiceinspectorates.gov.uk/hmiprisons/inspections/short-scrutiny-visit-aggregate-report/>

<https://www.justiceinspectorates.gov.uk/hmiprisons/inspections/hmyoi-feltham-a/>

<https://www.justiceinspectorates.gov.uk/hmiprisons/inspections/second-young-offender-institutions-short-scrutiny-visit/>

<https://www.justiceinspectorates.gov.uk/hmiprisons/inspections/hmyoi-parc-3/>

<https://www.justiceinspectorates.gov.uk/hmiprisons/inspections/short-scrutiny-visit-aggregate-report/>

<https://www.justiceinspectorates.gov.uk/hmiprisons/inspections/hmyoi-wetherby-and-keppel-4/>

<https://www.justiceinspectorates.gov.uk/hmiprisons/inspections/oakhill-secure-training-centre-9/>

<https://www.justiceinspectorates.gov.uk/hmiprisons/inspections/oakhill-secure-training-centre-10/>

<https://www.justiceinspectorates.gov.uk/hmiprisons/inspections/oakhill-secure-training-centre-11/>

<https://www.justiceinspectorates.gov.uk/hmiprisons/inspections/oakhill-secure-training-centre-12/>

<https://www.justiceinspectorates.gov.uk/hmiprisons/inspections/rainsbrook-secure-training-centre-8/>

<https://www.justiceinspectorates.gov.uk/hmiprisons/inspections/rainsbrook-secure-training-centre-9/>

<https://www.justiceinspectorates.gov.uk/hmiprisons/inspections/rainsbrook-secure-training-centre-10/>

<https://www.justiceinspectorates.gov.uk/hmiprisons/inspections/rainsbrook-secure-training-centre-12/>

<https://reports.ofsted.gov.uk/provider/2/SC033457> (See May 21 report)

<https://reports.ofsted.gov.uk/provider/2/SC046524> (See Mar 20 & Nov 20 reports)

<https://reports.ofsted.gov.uk/provider/2/SC035648> (See Nov 20 & Apr 21 reports)

<https://reports.ofsted.gov.uk/provider/2/SC040500> (See Aug 21 report)

<https://reports.ofsted.gov.uk/provider/2/SC036740> (See Sept 20 & Jun 21 reports)

<https://reports.ofsted.gov.uk/provider/2/SC042921> (See May 21 report)

<https://reports.ofsted.gov.uk/provider/2/SC035500> (See Sept 20, Apr 21 & Jun 21 reports)

## Pre COVID Inspection Reports

<https://www.justiceinspectorates.gov.uk/hmiprisons/inspections/hmyoi-cookham-wood-6/>

<https://www.justiceinspectorates.gov.uk/hmiprisons/inspections/hmyoi-feltham-a-childrens-unit-2/>

<https://www.justiceinspectorates.gov.uk/hmiprisons/inspections/hmyoi-parc-2/>

<https://www.justiceinspectorates.gov.uk/hmiprisons/inspections/hmyoi-werrington-6/>

<https://www.justiceinspectorates.gov.uk/hmiprisons/inspections/hmyoi-wetherby-and-keppel-3/>

<https://www.justiceinspectorates.gov.uk/hmiprisons/inspections/oakhill-secure-training-centre-8/>

<https://www.justiceinspectorates.gov.uk/hmiprisons/inspections/rainsbrook-secure-training-centre-7/>

<https://reports.ofsted.gov.uk/provider/2/SC033457> (See Jan 20 report)

<https://reports.ofsted.gov.uk/provider/2/SC046524> (See Dec 19 report)

<https://reports.ofsted.gov.uk/provider/2/SC035648> (See Feb 20 report)

<https://reports.ofsted.gov.uk/provider/2/SC040500> (See Jan 20 report)

<https://reports.ofsted.gov.uk/provider/2/SC036740> (See Oct 19 report)

<https://reports.ofsted.gov.uk/provider/2/SC042921> (See Feb 20 report)

<https://reports.ofsted.gov.uk/provider/2/SC035500> (See Jun 19 report)
