# Supplementary material for: The impact of the COVID-19 pandemic for children in custody: An analysis of inspection reports
Source: PLoS One. 2024 Jun 20;19(6):e0304862. doi: 10.1371/journal.pone.0304862 (PMC11189207; doi:10.1371/journal.pone.0304862)
Supplement: Attachment — Submitted filename: Response to Reviewers.docx [file pone.0304862.s002.docx]

Dr Charlotte Lennox

Jean McFarlane Building

Oxford Road

Manchester

M13 9PL

20/12/23

Dear Academic Editor,

Many thanks for the opportunity to submit a revised manuscript and to the reviewers for their helpful comments. Please find below all comments and how the authors have responded to them.

Journal requirements:

1. Please ensure that your manuscript meets PLOS ONE's style requirements, including those for file naming.

**The manuscript has been amended to meet PLOS ONE’s style requirements.**

2. Financial disclosure

**This has been included in the cover letter.**

2. Data availability

**This has been included in the cover letter.**

4. PLOS requires an ORCID iD for the corresponding author in Editorial Manager.

**This has been added.**

**Review Comments**

Reviewer #1: This is a straightforward, descriptive paper about the impact of Covid-19 on children in custody in the UK. The paper is well structured and well written albeit quite lengthy. I would recommend it is published and only have a few comments:

• By the time I had read to the end of the lengthy Findings section, I was unsure what the most important point was that the author team wanted to reader to take home. Likewise, the Abstract does not have a central main point pulling it all together. The paper is pitched as being about the mental health of children in custody but there was less emphasis on exploring this and significantly more descriptive content about the practicalities of Covid-19 restrictions and practices in child custodial settings. I think the author team should either a) slightly re-write elements of the paper to turn it into a paper that focuses more on process of Covid-19 restrictions or b) they should write a new section which firmly brings together all the findings to make clear statements about how Covid-19 impacted on children’s mental health.
**The introduction and elsewhere has been edited to remove the emphasis on mental health and more clearly highlight the focus on the impact of COVID-19 restrictions and practices. The results section has been reduced to focus more clearly on the impact of COVID-19 restrictions and practices.**

• I was unsure how the analytic framework was developed (line 206). Were these headings that are already in the inspection reports? If so, how did you allow for inductive findings to arise? If not, could you add in some detail about how the framework was developed?

**We have provided more clarity around the analysis and also tried to remove the confusion around the term framework in relation to the frameworks for the inspections vs. an analytical framework.**

**Reviewer #2:** While the findings of this study are of interest it is important that the authors redraft to address some major deficits before resubmitting.

The Discussion section is inadequate and contains too much that is in effect a summary of findings and not a discussion of the implications of those findings.

**The discussion has been rewritten to reflect the changes to the findings section but also to provide more discussion of the implications.**

Given that the article would be read by an international audience, a box summarising the agencies with their acronyms and responsibilities would be very useful if not essential.

**There is already a list of acronyms provided** **within the manuscript, therefore rather than duplicating this we have summarised the responsibilities within the manuscript and reduced the use of agencies for international audiences.**

Similarly some terms such as 'bubbles' took a special meaning during COVID and should be explained.

**These terms have been explained.**

I would advise the authors to avoid use of significant as the changes etc were not measurable and it introduces potential confusion.

**The use of the word significant has been removed throughout.**

Overall there are too many grammar/ punctuation errors which indicate the need for the article to be corrected before submission.

**These have been addressed.**

Yours sincerely

Charlotte Lennox
